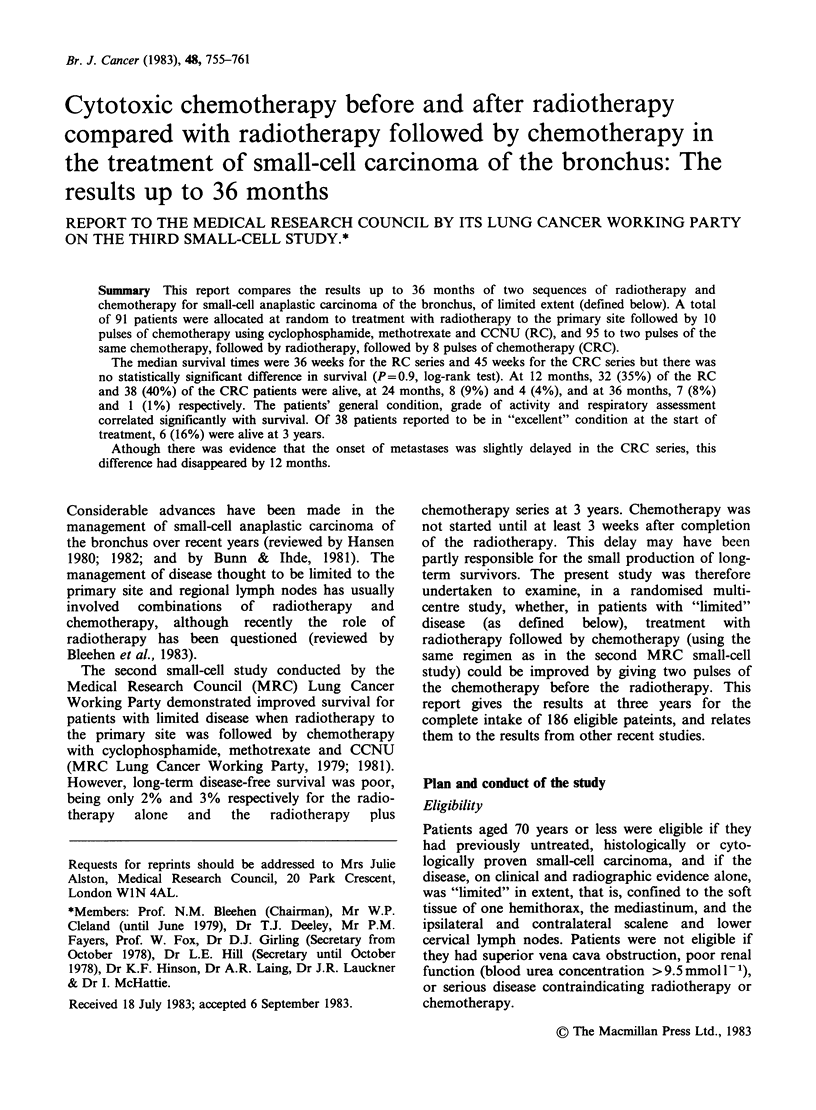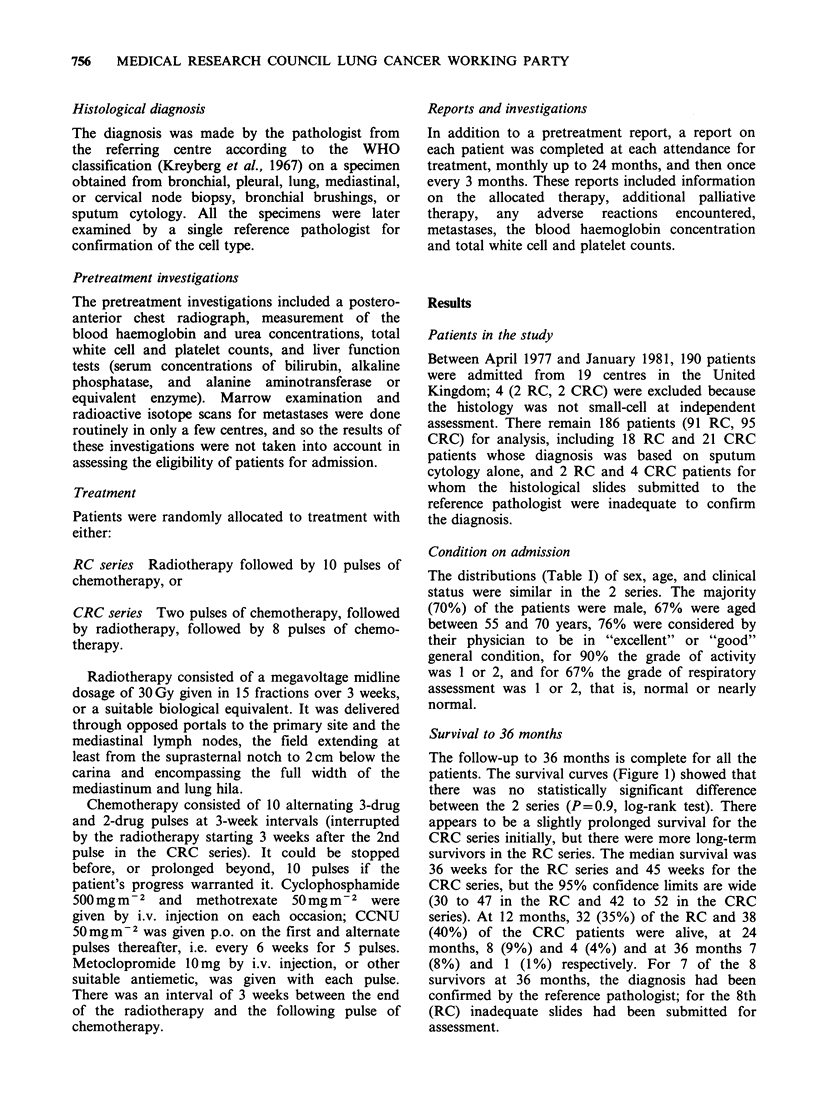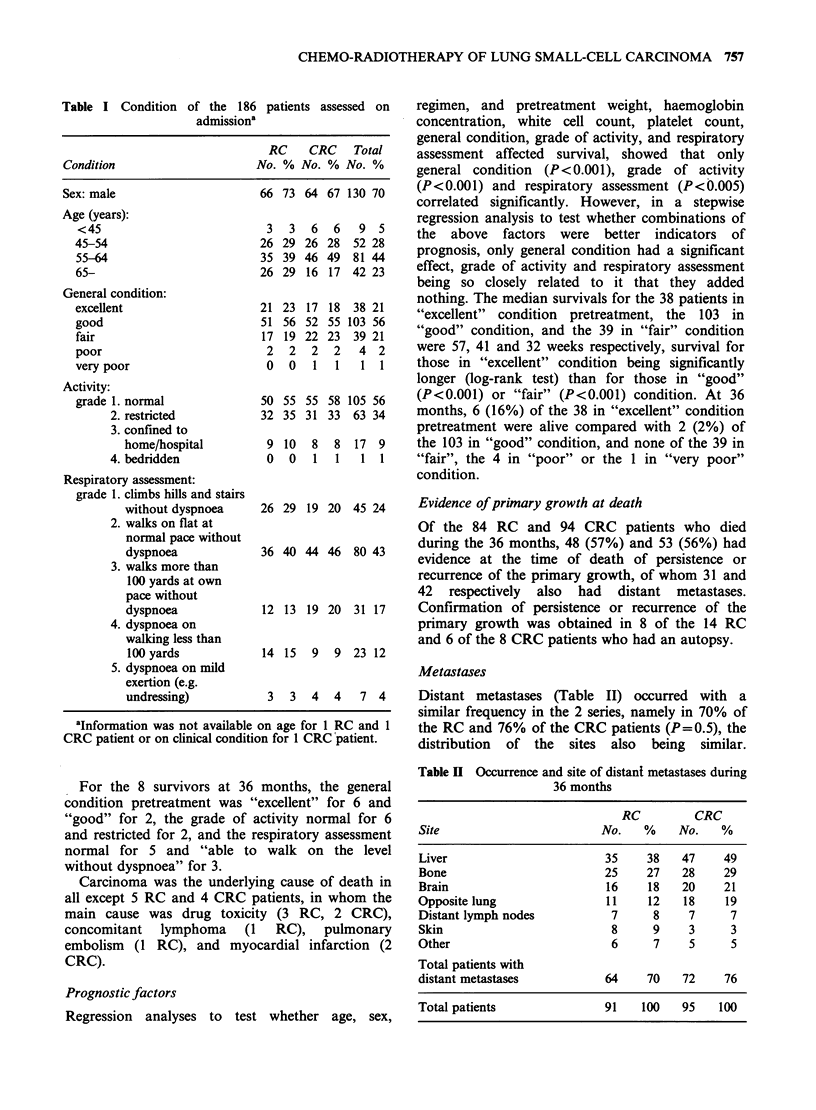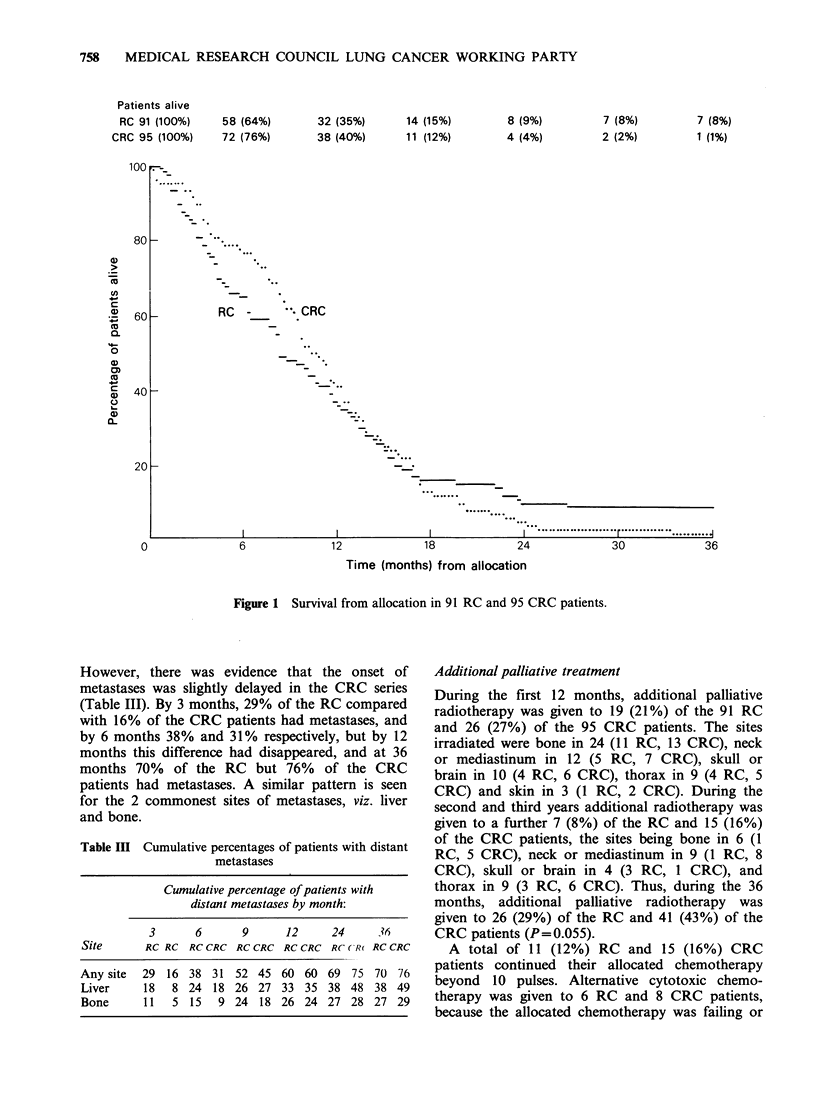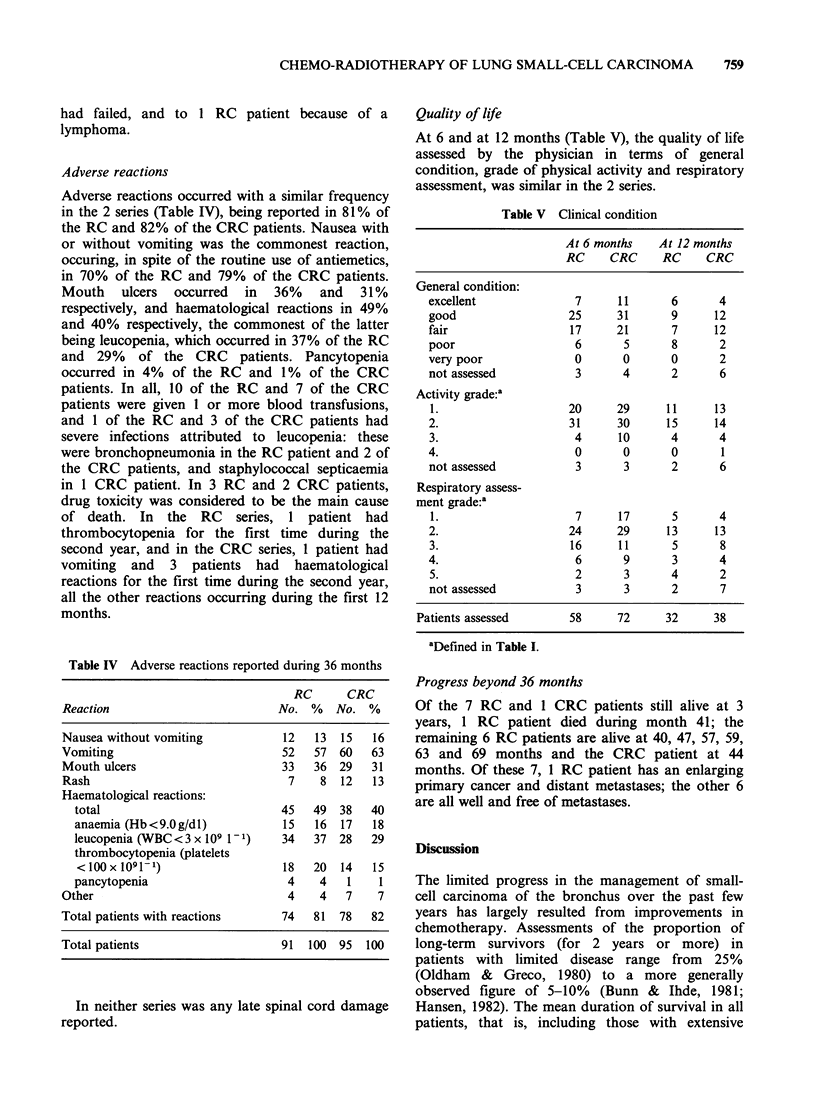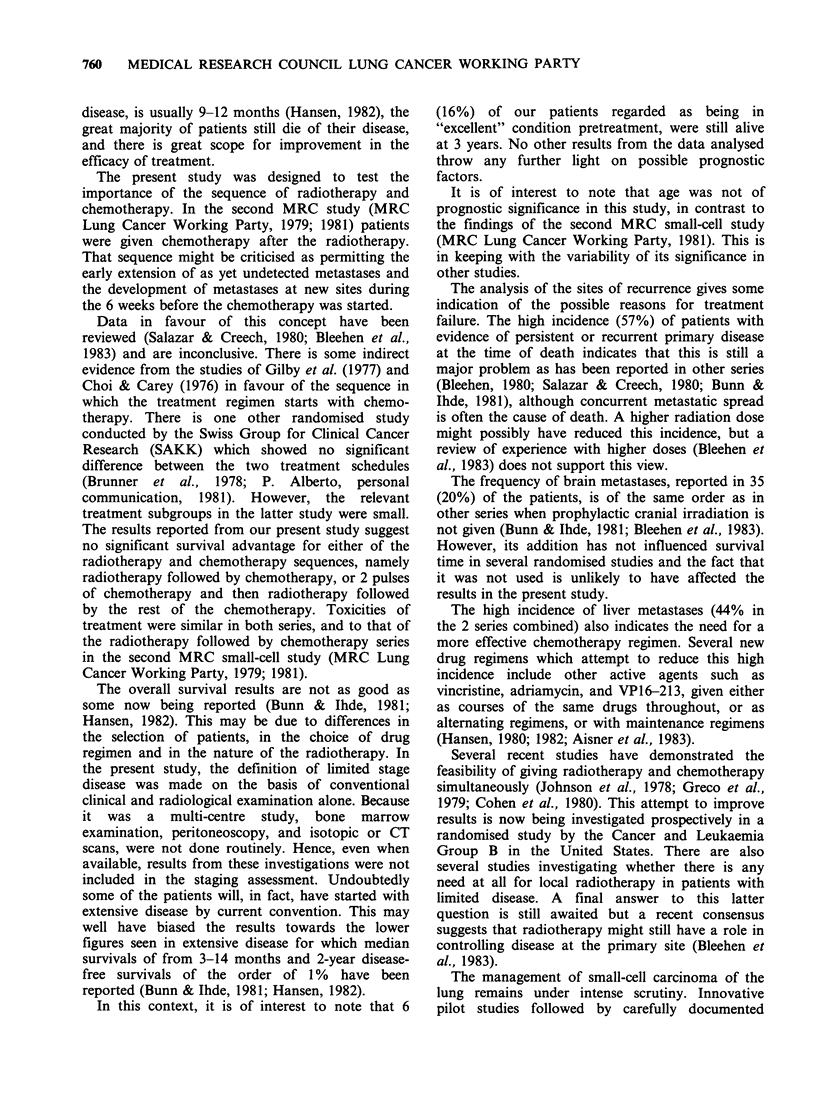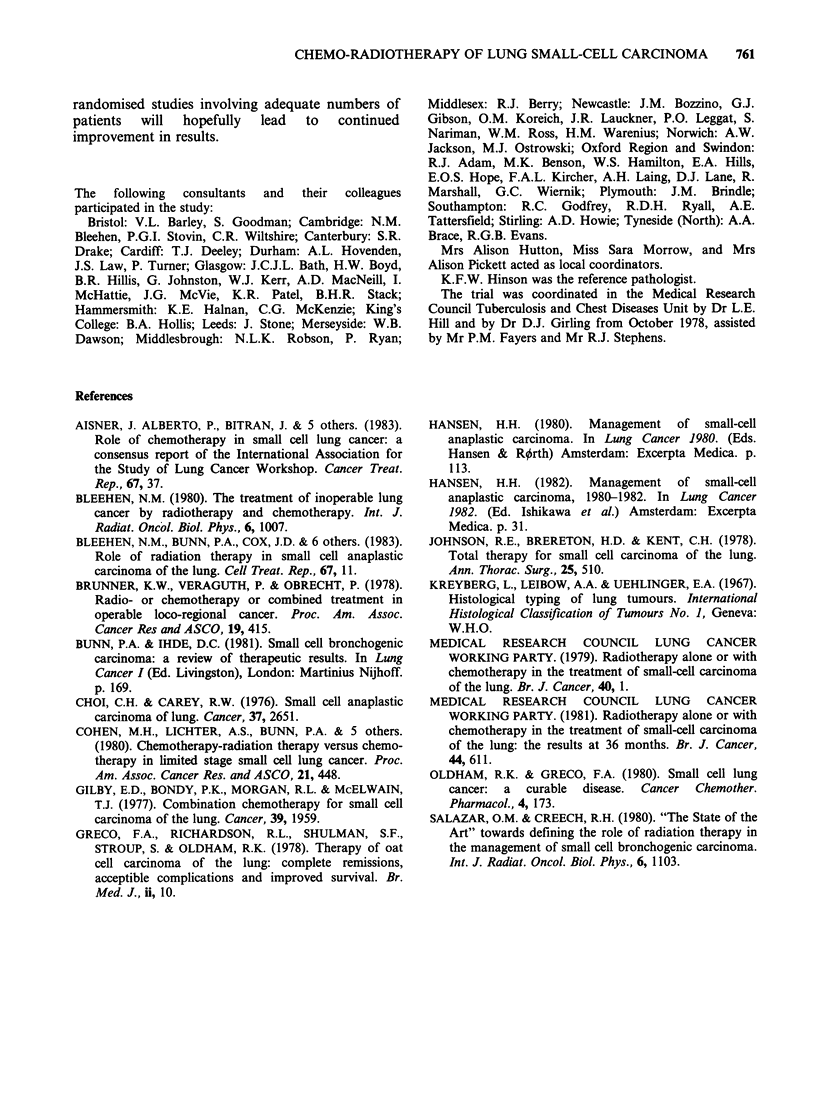# Cytotoxic chemotherapy before and after radiotherapy compared with radiotherapy followed by chemotherapy in the treatment of small-cell carcinoma of the bronchus: the results up to 36 months.

**DOI:** 10.1038/bjc.1983.264

**Published:** 1983-12

**Authors:** 

## Abstract

This report compares the results up to 36 months of two sequences of radiotherapy and chemotherapy for small-cell anaplastic carcinoma of the bronchus, of limited extent (defined below). A total of 91 patients were allocated at random to treatment with radiotherapy to the primary site followed by 10 pulses of chemotherapy using cyclophosphamide, methotrexate and CCNU (RC), and 95 to two pulses of the same chemotherapy, followed by radiotherapy, followed by 8 pulses of chemotherapy (CRC). The median survival times were 36 weeks for the RC series and 45 weeks for the CRC series but there was no statistically significant difference in survival (P = 0.9, log-rank test). At 12 months, 32 (35%) of the RC and 38 (40%) of the CRC patients were alive, at 24 months, 8 (9%) and 4 (4%), and at 36 months, 7 (8%) and 1 (1%) respectively. The patients' general condition, grade of activity and respiratory assessment correlated significantly with survival. Of 38 patients reported to be in "excellent" condition at the start of treatment, 6 (16%) were alive at 3 years. Although there was evidence that the onset of metastases was slightly delayed in the CRC series, this difference had disappeared by 12 months.


*Br. J. Cancer* (1983), **48**, 755–761

### Cytotoxic chemotherapy before and after radiotherapy compared with radiotherapy followed by chemotherapy in the treatment of small-cell carcinoma of the bronchus: The results up to 36 months

REPORT TO THE MEDICAL RESEARCH COUNCIL BY ITS LUNG CANCER WORKING PARTY ON THE THIRD SMALL-CELL STUDY.*

**Summary** This report compares the results up to 36 months of two sequences of radiotherapy and chemotherapy for small-cell anaplastic carcinoma of the bronchus, of limited extent (defined below). A total of 91 patients were allocated at random to treatment with radiotherapy to the primary site followed by 10 pulses of chemotherapy using cyclophosphamide, methotrexate and CCNU (RC), and 95 to two pulses of the same chemotherapy, followed by radiotherapy, followed by 8 pulses of chemotherapy (CRC).

The median survival times were 36 weeks for the RC series and 45 weeks for the CRC series but there was no statistically significant difference in survival ($P=0.9$, log-rank test). At 12 months, 32 (35%) of the RC and 38 (40%) of the CRC patients were alive, at 24 months, 8 (9%) and 4 (4%), and at 36 months, 7 (8%) and 1 (1%) respectively. The patients' general condition, grade of activity and respiratory assessment correlated significantly with survival. Of 38 patients reported to be in "excellent" condition at the start of treatment, 6 (16%) were alive at 3 years.

Athough there was evidence that the onset of metastases was slightly delayed in the CRC series, this difference had disappeared by 12 months.

Considerable advances have been made in the management of small-cell anaplastic carcinoma of the bronchus over recent years (reviewed by Hansen 1980; 1982; and by Bunn & Ihde, 1981). The management of disease thought to be limited to the primary site and regional lymph nodes has usually involved combinations of radiotherapy and chemotherapy, although recently the role of radiotherapy has been questioned (reviewed by Bleehen *et al.*, 1983).

The second small-cell study conducted by the Medical Research Council (MRC) Lung Cancer Working Party demonstrated improved survival for patients with limited disease when radiotherapy to the primary site was followed by chemotherapy with cyclophosphamide, methotrexate and CCNU (MRC Lung Cancer Working Party, 1979; 1981). However, long-term disease-free survival was poor, being only 2% and 3% respectively for the radiotherapy alone and the radiotherapy plus chemotherapy series at 3 years. Chemotherapy was not started until at least 3 weeks after completion of the radiotherapy. This delay may have been partly responsible for the small production of long-term survivors. The present study was therefore undertaken to examine, in a randomised multi-centre study, whether, in patients with "limited" disease (as defined below), treatment with radiotherapy followed by chemotherapy (using the same regimen as in the second MRC small-cell study) could be improved by giving two pulses of the chemotherapy before the radiotherapy. This report gives the results at three years for the complete intake of 186 eligible pateints, and relates them to the results from other recent studies.

### Plan and conduct of the study

### *Eligibility*

Patients aged 70 years or less were eligible if they had previously untreated, histologically or cytologically proven small-cell carcinoma, and if the disease, on clinical and radiographic evidence alone, was "limited" in extent, that is, confined to the soft tissue of one hemithorax, the mediastinum, and the ipsilateral and contralateral scalene and lower cervical lymph nodes. Patients were not eligible if they had superior vena cava obstruction, poor renal function (blood urea concentration $>9.5\,mmol\,l^{-1}$), or serious disease contraindicating radiotherapy or chemotherapy.

---

Requests for reprints should be addressed to Mrs Julie Alston, Medical Research Council, 20 Park Crescent, London W1N 4AL.

*Members: Prof. N.M. Bleehen (Chairman), Mr W.P. Cleland (until June 1979), Dr T.J. Deeley, Mr P.M. Fayers, Prof. W. Fox, Dr D.J. Girling (Secretary from October 1978), Dr L.E. Hill (Secretary until October 1978), Dr K.F. Hinson, Dr A.R. Laing, Dr J.R. Lauckner & Dr I. McHattie.

Received 18 July 1983; accepted 6 September 1983.

© The Macmillan Press Ltd., 1983

*Histological diagnosis*

The diagnosis was made by the pathologist from the referring centre according to the WHO classification (Kreyberg *et al.*, 1967) on a specimen obtained from bronchial, pleural, lung, mediastinal, or cervical node biopsy, bronchial brushings, or sputum cytology. All the specimens were later examined by a single reference pathologist for confirmation of the cell type.

*Pretreatment investigations*

The pretreatment investigations included a postero-anterior chest radiograph, measurement of the blood haemoglobin and urea concentrations, total white cell and platelet counts, and liver function tests (serum concentrations of bilirubin, alkaline phosphatase, and alanine aminotransferase or equivalent enzyme). Marrow examination and radioactive isotope scans for metastases were done routinely in only a few centres, and so the results of these investigations were not taken into account in assessing the eligibility of patients for admission.

*Treatment*

Patients were randomly allocated to treatment with either:

*RC series* Radiotherapy followed by 10 pulses of chemotherapy, or

*CRC series* Two pulses of chemotherapy, followed by radiotherapy, followed by 8 pulses of chemotherapy.

Radiotherapy consisted of a megavoltage midline dosage of 30 Gy given in 15 fractions over 3 weeks, or a suitable biological equivalent. It was delivered through opposed portals to the primary site and the mediastinal lymph nodes, the field extending at least from the suprasternal notch to 2 cm below the carina and encompassing the full width of the mediastinum and lung hila.

Chemotherapy consisted of 10 alternating 3-drug and 2-drug pulses at 3-week intervals (interrupted by the radiotherapy starting 3 weeks after the 2nd pulse in the CRC series). It could be stopped before, or prolonged beyond, 10 pulses if the patient's progress warranted it. Cyclophosphamide $500\,mg\,m^{-2}$ and methotrexate $50\,mg\,m^{-2}$ were given by i.v. injection on each occasion; CCNU $50\,mg\,m^{-2}$ was given p.o. on the first and alternate pulses thereafter, i.e. every 6 weeks for 5 pulses. Metoclopromide 10 mg by i.v. injection, or other suitable antiemetic, was given with each pulse. There was an interval of 3 weeks between the end of the radiotherapy and the following pulse of chemotherapy.

*Reports and investigations*

In addition to a pretreatment report, a report on each patient was completed at each attendance for treatment, monthly up to 24 months, and then once every 3 months. These reports included information on the allocated therapy, additional palliative therapy, any adverse reactions encountered, metastases, the blood haemoglobin concentration and total white cell and platelet counts.

### Results

*Patients in the study*

Between April 1977 and January 1981, 190 patients were admitted from 19 centres in the United Kingdom; 4 (2 RC, 2 CRC) were excluded because the histology was not small-cell at independent assessment. There remain 186 patients (91 RC, 95 CRC) for analysis, including 18 RC and 21 CRC patients whose diagnosis was based on sputum cytology alone, and 2 RC and 4 CRC patients for whom the histological slides submitted to the reference pathologist were inadequate to confirm the diagnosis.

*Condition on admission*

The distributions (Table I) of sex, age, and clinical status were similar in the 2 series. The majority (70%) of the patients were male, 67% were aged between 55 and 70 years, 76% were considered by their physician to be in "excellent" or "good" general condition, for 90% the grade of activity was 1 or 2, and for 67% the grade of respiratory assessment was 1 or 2, that is, normal or nearly normal.

*Survival to 36 months*

The follow-up to 36 months is complete for all the patients. The survival curves (Figure 1) showed that there was no statistically significant difference between the 2 series ($P=0.9$, log-rank test). There appears to be a slightly prolonged survival for the CRC series initially, but there were more long-term survivors in the RC series. The median survival was 36 weeks for the RC series and 45 weeks for the CRC series, but the 95% confidence limits are wide (30 to 47 in the RC and 42 to 52 in the CRC series). At 12 months, 32 (35%) of the RC and 38 (40%) of the CRC patients were alive, at 24 months, 8 (9%) and 4 (4%) and at 36 months 7 (8%) and 1 (1%) respectively. For 7 of the 8 survivors at 36 months, the diagnosis had been confirmed by the reference pathologist; for the 8th (RC) inadequate slides had been submitted for assessment.

**Table I** Condition of the 186 patients assessed on admission[a]

| *Condition* | *RC* *No.* | *%* | *CRC* *No.* | *%* | *Total* *No.* | *%* |
|---|---|---|---|---|---|---|
| Sex: male | 66 | 73 | 64 | 67 | 130 | 70 |
| Age (years): | | | | | | |
| <45 | 3 | 3 | 6 | 6 | 9 | 5 |
| 45–54 | 26 | 29 | 26 | 28 | 52 | 28 |
| 55–64 | 35 | 39 | 46 | 49 | 81 | 44 |
| 65– | 26 | 29 | 16 | 17 | 42 | 23 |
| General condition: | | | | | | |
| excellent | 21 | 23 | 17 | 18 | 38 | 21 |
| good | 51 | 56 | 52 | 55 | 103 | 56 |
| fair | 17 | 19 | 22 | 23 | 39 | 21 |
| poor | 2 | 2 | 2 | 2 | 4 | 2 |
| very poor | 0 | 0 | 1 | 1 | 1 | 1 |
| Activity: | | | | | | |
| grade 1. normal | 50 | 55 | 55 | 58 | 105 | 56 |
| 2. restricted | 32 | 35 | 31 | 33 | 63 | 34 |
| 3. confined to home/hospital | 9 | 10 | 8 | 8 | 17 | 9 |
| 4. bedridden | 0 | 0 | 1 | 1 | 1 | 1 |
| Respiratory assessment: | | | | | | |
| grade 1. climbs hills and stairs without dyspnoea | 26 | 29 | 19 | 20 | 45 | 24 |
| 2. walks on flat at normal pace without dyspnoea | 36 | 40 | 44 | 46 | 80 | 43 |
| 3. walks more than 100 yards at own pace without dyspnoea | 12 | 13 | 19 | 20 | 31 | 17 |
| 4. dyspnoea on walking less than 100 yards | 14 | 15 | 9 | 9 | 23 | 12 |
| 5. dyspnoea on mild exertion (e.g. undressing) | 3 | 3 | 4 | 4 | 7 | 4 |

[a]Information was not available on age for 1 RC and 1 CRC patient or on clinical condition for 1 CRC patient.

For the 8 survivors at 36 months, the general condition pretreatment was "excellent" for 6 and "good" for 2, the grade of activity normal for 6 and restricted for 2, and the respiratory assessment normal for 5 and "able to walk on the level without dyspnoea" for 3.

Carcinoma was the underlying cause of death in all except 5 RC and 4 CRC patients, in whom the main cause was drug toxicity (3 RC, 2 CRC), concomitant lymphoma (1 RC), pulmonary embolism (1 RC), and myocardial infarction (2 CRC).

*Prognostic factors*

Regression analyses to test whether age, sex, regimen, and pretreatment weight, haemoglobin concentration, white cell count, platelet count, general condition, grade of activity, and respiratory assessment affected survival, showed that only general condition ($P<0.001$), grade of activity ($P<0.001$) and respiratory assessment ($P<0.005$) correlated significantly. However, in a stepwise regression analysis to test whether combinations of the above factors were better indicators of prognosis, only general condition had a significant effect, grade of activity and respiratory assessment being so closely related to it that they added nothing. The median survivals for the 38 patients in "excellent" condition pretreatment, the 103 in "good" condition, and the 39 in "fair" condition were 57, 41 and 32 weeks respectively, survival for those in "excellent" condition being significantly longer (log-rank test) than for those in "good" ($P<0.001$) or "fair" ($P<0.001$) condition. At 36 months, 6 (16%) of the 38 in "excellent" condition pretreatment were alive compared with 2 (2%) of the 103 in "good" condition, and none of the 39 in "fair", the 4 in "poor" or the 1 in "very poor" condition.

*Evidence of primary growth at death*

Of the 84 RC and 94 CRC patients who died during the 36 months, 48 (57%) and 53 (56%) had evidence at the time of death of persistence or recurrence of the primary growth, of whom 31 and 42 respectively also had distant metastases. Confirmation of persistence or recurrence of the primary growth was obtained in 8 of the 14 RC and 6 of the 8 CRC patients who had an autopsy.

*Metastases*

Distant metastases (Table II) occurred with a similar frequency in the 2 series, namely in 70% of the RC and 76% of the CRC patients ($P=0.5$), the distribution of the sites also being similar.

**Table II** Occurrence and site of distant metastases during 36 months

| *Site* | *RC* *No.* | *%* | *CRC* *No.* | *%* |
|---|---|---|---|---|
| Liver | 35 | 38 | 47 | 49 |
| Bone | 25 | 27 | 28 | 29 |
| Brain | 16 | 18 | 20 | 21 |
| Opposite lung | 11 | 12 | 18 | 19 |
| Distant lymph nodes | 7 | 8 | 7 | 7 |
| Skin | 8 | 9 | 3 | 3 |
| Other | 6 | 7 | 5 | 5 |
| Total patients with distant metastases | 64 | 70 | 72 | 76 |
| Total patients | 91 | 100 | 95 | 100 |

**Figure 1** Survival from allocation in 91 RC and 95 CRC patients.

However, there was evidence that the onset of metastases was slightly delayed in the CRC series (Table III). By 3 months, 29% of the RC compared with 16% of the CRC patients had metastases, and by 6 months 38% and 31% respectively, but by 12 months this difference had disappeared, and at 36 months 70% of the RC but 76% of the CRC patients had metastases. A similar pattern is seen for the 2 commonest sites of metastases, *viz.* liver and bone.

**Table III** Cumulative percentages of patients with distant metastases

| | *Cumulative percentage of patients with distant metastases by month:* | | | | | | | | | | | |
|---|---|---|---|---|---|---|---|---|---|---|---|---|
| | *3* | | *6* | | *9* | | *12* | | *24* | | *36* | |
| *Site* | *RC* | *RC* | *RC* | *CRC* | *RC* | *CRC* | *RC* | *CRC* | *RC* | *CRC* | *RC* | *CRC* |
| Any site | 29 | 16 | 38 | 31 | 52 | 45 | 60 | 60 | 69 | 75 | 70 | 76 |
| Liver | 18 | 8 | 24 | 18 | 26 | 27 | 33 | 35 | 38 | 48 | 38 | 49 |
| Bone | 11 | 5 | 15 | 9 | 24 | 18 | 26 | 24 | 27 | 28 | 27 | 29 |

*Additional palliative treatment*

During the first 12 months, additional palliative radiotherapy was given to 19 (21%) of the 91 RC and 26 (27%) of the 95 CRC patients. The sites irradiated were bone in 24 (11 RC, 13 CRC), neck or mediastinum in 12 (5 RC, 7 CRC), skull or brain in 10 (4 RC, 6 CRC), thorax in 9 (4 RC, 5 CRC) and skin in 3 (1 RC, 2 CRC). During the second and third years additional radiotherapy was given to a further 7 (8%) of the RC and 15 (16%) of the CRC patients, the sites being bone in 6 (1 RC, 5 CRC), neck or mediastinum in 9 (1 RC, 8 CRC), skull or brain in 4 (3 RC, 1 CRC), and thorax in 9 (3 RC, 6 CRC). Thus, during the 36 months, additional palliative radiotherapy was given to 26 (29%) of the RC and 41 (43%) of the CRC patients ($P = 0.055$).

A total of 11 (12%) RC and 15 (16%) CRC patients continued their allocated chemotherapy beyond 10 pulses. Alternative cytotoxic chemotherapy was given to 6 RC and 8 CRC patients, because the allocated chemotherapy was failing or had failed, and to 1 RC patient because of a lymphoma.

*Adverse reactions*

Adverse reactions occurred with a similar frequency in the 2 series (Table IV), being reported in 81% of the RC and 82% of the CRC patients. Nausea with or without vomiting was the commonest reaction, occuring, in spite of the routine use of antiemetics, in 70% of the RC and 79% of the CRC patients. Mouth ulcers occurred in 36% and 31% respectively, and haematological reactions in 49% and 40% respectively, the commonest of the latter being leucopenia, which occurred in 37% of the RC and 29% of the CRC patients. Pancytopenia occurred in 4% of the RC and 1% of the CRC patients. In all, 10 of the RC and 7 of the CRC patients were given 1 or more blood transfusions, and 1 of the RC and 3 of the CRC patients had severe infections attributed to leucopenia: these were bronchopneumonia in the RC patient and 2 of the CRC patients, and staphylococcal septicaemia in 1 CRC patient. In 3 RC and 2 CRC patients, drug toxicity was considered to be the main cause of death. In the RC series, 1 patient had thrombocytopenia for the first time during the second year, and in the CRC series, 1 patient had vomiting and 3 patients had haematological reactions for the first time during the second year, all the other reactions occurring during the first 12 months.

**Table IV** Adverse reactions reported during 36 months

| *Reaction* | *RC No.* | *RC %* | *CRC No.* | *CRC %* |
|---|---|---|---|---|
| Nausea without vomiting | 12 | 13 | 15 | 16 |
| Vomiting | 52 | 57 | 60 | 63 |
| Mouth ulcers | 33 | 36 | 29 | 31 |
| Rash | 7 | 8 | 12 | 13 |
| Haematological reactions: | | | | |
| total | 45 | 49 | 38 | 40 |
| anaemia (Hb < 9.0 g/dl) | 15 | 16 | 17 | 18 |
| leucopenia (WBC < $3 \times 10^9\ l^{-1}$) | 34 | 37 | 28 | 29 |
| thrombocytopenia (platelets < $100 \times 10^9 l^{-1}$) | 18 | 20 | 14 | 15 |
| pancytopenia | 4 | 4 | 1 | 1 |
| Other | 4 | 4 | 7 | 7 |
| Total patients with reactions | 74 | 81 | 78 | 82 |
| Total patients | 91 | 100 | 95 | 100 |

In neither series was any late spinal cord damage reported.

*Quality of life*

At 6 and at 12 months (Table V), the quality of life assessed by the physician in terms of general condition, grade of physical activity and respiratory assessment, was similar in the 2 series.

**Table V** Clinical condition

| | *At 6 months RC* | *At 6 months CRC* | *At 12 months RC* | *At 12 months CRC* |
|---|---|---|---|---|
| General condition: | | | | |
| excellent | 7 | 11 | 6 | 4 |
| good | 25 | 31 | 9 | 12 |
| fair | 17 | 21 | 7 | 12 |
| poor | 6 | 5 | 8 | 2 |
| very poor | 0 | 0 | 0 | 2 |
| not assessed | 3 | 4 | 2 | 6 |
| Activity grade:[a] | | | | |
| 1. | 20 | 29 | 11 | 13 |
| 2. | 31 | 30 | 15 | 14 |
| 3. | 4 | 10 | 4 | 4 |
| 4. | 0 | 0 | 0 | 1 |
| not assessed | 3 | 3 | 2 | 6 |
| Respiratory assessment grade:[a] | | | | |
| 1. | 7 | 17 | 5 | 4 |
| 2. | 24 | 29 | 13 | 13 |
| 3. | 16 | 11 | 5 | 8 |
| 4. | 6 | 9 | 3 | 4 |
| 5. | 2 | 3 | 4 | 2 |
| not assessed | 3 | 3 | 2 | 7 |
| Patients assessed | 58 | 72 | 32 | 38 |

[a]Defined in **Table I**.

*Progress beyond 36 months*

Of the 7 RC and 1 CRC patients still alive at 3 years, 1 RC patient died during month 41; the remaining 6 RC patients are alive at 40, 47, 57, 59, 63 and 69 months and the CRC patient at 44 months. Of these 7, 1 RC patient has an enlarging primary cancer and distant metastases; the other 6 are all well and free of metastases.

### Discussion

The limited progress in the management of small-cell carcinoma of the bronchus over the past few years has largely resulted from improvements in chemotherapy. Assessments of the proportion of long-term survivors (for 2 years or more) in patients with limited disease range from 25% (Oldham & Greco, 1980) to a more generally observed figure of 5–10% (Bunn & Ihde, 1981; Hansen, 1982). The mean duration of survival in all patients, that is, including those with extensive disease, is usually 9–12 months (Hansen, 1982), the great majority of patients still die of their disease, and there is great scope for improvement in the efficacy of treatment.

The present study was designed to test the importance of the sequence of radiotherapy and chemotherapy. In the second MRC study (MRC Lung Cancer Working Party, 1979; 1981) patients were given chemotherapy after the radiotherapy. That sequence might be criticised as permitting the early extension of as yet undetected metastases and the development of metastases at new sites during the 6 weeks before the chemotherapy was started.

Data in favour of this concept have been reviewed (Salazar & Creech, 1980; Bleehen *et al.*, 1983) and are inconclusive. There is some indirect evidence from the studies of Gilby *et al.* (1977) and Choi & Carey (1976) in favour of the sequence in which the treatment regimen starts with chemotherapy. There is one other randomised study conducted by the Swiss Group for Clinical Cancer Research (SAKK) which showed no significant difference between the two treatment schedules (Brunner *et al.*, 1978; P. Alberto, personal communication, 1981). However, the relevant treatment subgroups in the latter study were small. The results reported from our present study suggest no significant survival advantage for either of the radiotherapy and chemotherapy sequences, namely radiotherapy followed by chemotherapy, or 2 pulses of chemotherapy and then radiotherapy followed by the rest of the chemotherapy. Toxicities of treatment were similar in both series, and to that of the radiotherapy followed by chemotherapy series in the second MRC small-cell study (MRC Lung Cancer Working Party, 1979; 1981).

The overall survival results are not as good as some now being reported (Bunn & Ihde, 1981; Hansen, 1982). This may be due to differences in the selection of patients, in the choice of drug regimen and in the nature of the radiotherapy. In the present study, the definition of limited stage disease was made on the basis of conventional clinical and radiological examination alone. Because it was a multi-centre study, bone marrow examination, peritoneoscopy, and isotopic or CT scans, were not done routinely. Hence, even when available, results from these investigations were not included in the staging assessment. Undoubtedly some of the patients will, in fact, have started with extensive disease by current convention. This may well have biased the results towards the lower figures seen in extensive disease for which median survivals of from 3–14 months and 2-year disease-free survivals of the order of 1% have been reported (Bunn & Ihde, 1981; Hansen, 1982).

In this context, it is of interest to note that 6 (16%) of our patients regarded as being in "excellent" condition pretreatment, were still alive at 3 years. No other results from the data analysed throw any further light on possible prognostic factors.

It is of interest to note that age was not of prognostic significance in this study, in contrast to the findings of the second MRC small-cell study (MRC Lung Cancer Working Party, 1981). This is in keeping with the variability of its significance in other studies.

The analysis of the sites of recurrence gives some indication of the possible reasons for treatment failure. The high incidence (57%) of patients with evidence of persistent or recurrent primary disease at the time of death indicates that this is still a major problem as has been reported in other series (Bleehen, 1980; Salazar & Creech, 1980; Bunn & Ihde, 1981), although concurrent metastatic spread is often the cause of death. A higher radiation dose might possibly have reduced this incidence, but a review of experience with higher doses (Bleehen *et al.*, 1983) does not support this view.

The frequency of brain metastases, reported in 35 (20%) of the patients, is of the same order as in other series when prophylactic cranial irradiation is not given (Bunn & Ihde, 1981; Bleehen *et al.*, 1983). However, its addition has not influenced survival time in several randomised studies and the fact that it was not used is unlikely to have affected the results in the present study.

The high incidence of liver metastases (44% in the 2 series combined) also indicates the need for a more effective chemotherapy regimen. Several new drug regimens which attempt to reduce this high incidence include other active agents such as vincristine, adriamycin, and VP16–213, given either as courses of the same drugs throughout, or as alternating regimens, or with maintenance regimens (Hansen, 1980; 1982; Aisner *et al.*, 1983).

Several recent studies have demonstrated the feasibility of giving radiotherapy and chemotherapy simultaneously (Johnson *et al.*, 1978; Greco *et al.*, 1979; Cohen *et al.*, 1980). This attempt to improve results is now being investigated prospectively in a randomised study by the Cancer and Leukaemia Group B in the United States. There are also several studies investigating whether there is any need at all for local radiotherapy in patients with limited disease. A final answer to this latter question is still awaited but a recent consensus suggests that radiotherapy might still have a role in controlling disease at the primary site (Bleehen *et al.*, 1983).

The management of small-cell carcinoma of the lung remains under intense scrutiny. Innovative pilot studies followed by carefully documented randomised studies involving adequate numbers of patients will hopefully lead to continued improvement in results.

The following consultants and their colleagues participated in the study:

Bristol: V.L. Barley, S. Goodman; Cambridge: N.M. Bleehen, P.G.I. Stovin, C.R. Wiltshire; Canterbury: S.R. Drake; Cardiff: T.J. Deeley; Durham: A.L. Hovenden, J.S. Law, P. Turner; Glasgow: J.C.J.L. Bath, H.W. Boyd, B.R. Hillis, G. Johnston, W.J. Kerr, A.D. MacNeill, I. McHattie, J.G. McVie, K.R. Patel, B.H.R. Stack; Hammersmith: K.E. Halnan, C.G. McKenzie; King's College: B.A. Hollis; Leeds: J. Stone; Merseyside: W.B. Dawson; Middlesbrough: N.L.K. Robson, P. Ryan; Middlesex: R.J. Berry; Newcastle: J.M. Bozzino, G.J. Gibson, O.M. Koreich, J.R. Lauckner, P.O. Leggat, S. Nariman, W.M. Ross, H.M. Warenius; Norwich: A.W. Jackson, M.J. Ostrowski; Oxford Region and Swindon: R.J. Adam, M.K. Benson, W.S. Hamilton, E.A. Hills, E.O.S. Hope, F.A.L. Kircher, A.H. Laing, D.J. Lane, R. Marshall, G.C. Wiernik; Plymouth: J.M. Brindle; Southampton: R.C. Godfrey, R.D.H. Ryall, A.E. Tattersfield; Stirling: A.D. Howie; Tyneside (North): A.A. Brace, R.G.B. Evans.

Mrs Alison Hutton, Miss Sara Morrow, and Mrs Alison Pickett acted as local coordinators.

K.F.W. Hinson was the reference pathologist.

The trial was coordinated in the Medical Research Council Tuberculosis and Chest Diseases Unit by Dr L.E. Hill and by Dr D.J. Girling from October 1978, assisted by Mr P.M. Fayers and Mr R.J. Stephens.

### References

AISNER, J. ALBERTO, P., BITRAN, J. & 5 others. (1983). Role of chemotherapy in small cell lung cancer: a consensus report of the International Association for the Study of Lung Cancer Workshop. *Cancer Treat. Rep.*, **67**, 37.

BLEEHEN, N.M. (1980). The treatment of inoperable lung cancer by radiotherapy and chemotherapy. *Int. J. Radiat. Oncol. Biol. Phys.*, **6**, 1007.

BLEEHEN, N.M., BUNN, P.A., COX, J.D. & 6 others. (1983). Role of radiation therapy in small cell anaplastic carcinoma of the lung. *Cell Treat. Rep.*, **67**, 11.

BRUNNER, K.W., VERAGUTH, P. & OBRECHT, P. (1978). Radio- or chemotherapy or combined treatment in operable loco-regional cancer. *Proc. Am. Assoc. Cancer Res and ASCO*, **19**, 415.

BUNN, P.A. & IHDE, D.C. (1981). Small cell bronchogenic carcinoma: a review of therapeutic results. In *Lung Cancer I* (Ed. Livingston), London: Martinius Nijhoff. p. 169.

CHOI, C.H. & CAREY, R.W. (1976). Small cell anaplastic carcinoma of lung. *Cancer*, **37**, 2651.

COHEN, M.H., LICHTER, A.S., BUNN, P.A. & 5 others. (1980). Chemotherapy-radiation therapy versus chemotherapy in limited stage small cell lung cancer. *Proc. Am. Assoc. Cancer Res. and ASCO*, **21**, 448.

GILBY, E.D., BONDY, P.K., MORGAN, R.L. & McELWAIN, T.J. (1977). Combination chemotherapy for small cell carcinoma of the lung. *Cancer*, **39**, 1959.

GRECO, F.A., RICHARDSON, R.L., SHULMAN, S.F., STROUP, S. & OLDHAM, R.K. (1978). Therapy of oat cell carcinoma of the lung: complete remissions, acceptible complications and improved survival. *Br. Med. J.*, **ii**, 10.

HANSEN, H.H. (1980). Management of small-cell anaplastic carcinoma. In *Lung Cancer 1980*. (Eds. Hansen & Rørth) Amsterdam: Excerpta Medica. p. 113.

HANSEN, H.H. (1982). Management of small-cell anaplastic carcinoma, 1980–1982. In *Lung Cancer 1982*. (Ed. Ishikawa *et al.*) Amsterdam: Excerpta Medica. p. 31.

JOHNSON, R.E., BRERETON, H.D. & KENT, C.H. (1978). Total therapy for small cell carcinoma of the lung. *Ann. Thorac. Surg.*, **25**, 510.

KREYBERG, L., LEIBOW, A.A. & UEHLINGER, E.A. (1967). Histological typing of lung tumours. *International Histological Classification of Tumours No. 1*, Geneva: W.H.O.

MEDICAL RESEARCH COUNCIL LUNG CANCER WORKING PARTY. (1979). Radiotherapy alone or with chemotherapy in the treatment of small-cell carcinoma of the lung. *Br. J. Cancer*, **40**, 1.

MEDICAL RESEARCH COUNCIL LUNG CANCER WORKING PARTY. (1981). Radiotherapy alone or with chemotherapy in the treatment of small-cell carcinoma of the lung: the results at 36 months. *Br. J. Cancer*, **44**, 611.

OLDHAM, R.K. & GRECO, F.A. (1980). Small cell lung cancer: a curable disease. *Cancer Chemother. Pharmacol.*, **4**, 173.

SALAZAR, O.M. & CREECH, R.H. (1980). "The State of the Art" towards defining the role of radiation therapy in the management of small cell bronchogenic carcinoma. *Int. J. Radiat. Oncol. Biol. Phys.*, **6**, 1103.